# m^6^A RNA methylation regulators contribute to malignant progression and have clinical prognostic impact in gliomas

**DOI:** 10.18632/aging.101829

**Published:** 2019-02-27

**Authors:** Rui-Chao Chai, Fan Wu, Qi-Xue Wang, Shu Zhang, Ke-Nan Zhang, Yu-Qing Liu, Zheng Zhao, Tao Jiang, Yong-Zhi Wang, Chun-Sheng Kang

**Affiliations:** 1 Department of Molecular Neuropathology, Beijing Neurosurgical Institute, Beijing Tiantan Hospital, Capital Medical University, Beijing 100160, China; 2 Department of Neurosurgery, Beijing Tiantan Hospital, Capital Medical University, Beijing 100160, China; 3 China National Clinical Research Center for Neurological Diseases, Beijing, China; 4 Laboratory of Neuro-Oncology, Tianjin Neurological Institute, Department of Neurosurgery, Tianjin Medical University General Hospital and Key Laboratory of Neurotrauma, Variation, and Regeneration, Ministry of Education and Tianjin Municipal Government, Tianjin 300052, China; 5 Chinese Glioma Genome Atlas Network (CGGA); 6 Affiliated Cancer Hospital & Institute of Guangzhou Medical University, Guangzhou 510095, China; * Equal contribution

**Keywords:** RNA modification, methyltransferase, demethylases, epigenetics, prognostic signature

## Abstract

N6-methyladenosine (m^6^A) RNA methylation, associated with cancer initiation and progression, is dynamically regulated by the m^6^A RNA methylation regulators (“writers”, “erasers” and “readers”). Here, we demonstrate that most of the thirteen main m^6^A RNA methylation regulators are differentially expressed among gliomas stratified by different clinicopathological features in 904 gliomas. We identified two subgroups of gliomas (RM1/2) by applying consensus clustering to m^6^A RNA methylation regulators. Compared with the RM1 subgroup, the RM2 subgroup correlates with a poorer prognosis, higher WHO grade, and lower frequency of *IDH* mutation. Moreover, the hallmarks of epithelial-mesenchymal transition and TNFα signaling via NF-κB are also significantly enriched in the RM2 subgroup. This finding indicates that m^6^A RNA methylation regulators are closely associated with glioma malignancy. Based on this finding, we derived a risk signature, using seven m^6^A RNA methylation regulators, that is not only an independent prognostic marker but can also predict the clinicopathological features of gliomas. Moreover, m^6^A regulators are associated with the mesenchymal subtype and TMZ sensitivity in GBM. In conclusion, m^6^A RNA methylation regulators are crucial participants in the malignant progression of gliomas and are potentially useful for prognostic stratification and treatment strategy development.

## Introduction

In the traditional concept, epigenetics only involves the reversible chemical modification of DNA or proteins (histones) and regulates gene expression independent of DNA sequences, which could be heritable through cell division [1]. RNA modification was first discovered in the 1970s, but it has only recently been considered a third layer of epigenetics, along with the emerging functions of regulating RNA processing and metabolism [1–7]. Currently, RNA modification has been identified in almost all forms of native cellular RNA, including mRNAs, tRNAs, rRNAs, small nuclear RNAs, small nucleolar RNAs, long noncoding RNAs (lncRNAs) and micro-RNAs (miRNA) [5–12]. It has been reported in several forms, including N1-methyladenosine, N7-methyladenosine, 5-methylcytosine, pseudouridine, N6,2’-O-dimethyladenosine (m^6^A) and 2′-O-methylation [5,11,13]. Among these, the m^6^A modification was the first identified and is the most abundant form of mRNA methylation in eukaryotes [11,14,15].

RNA modification, similar to DNA and protein modification, is dynamically regulated by methyltransferases (‘writers’), binding proteins (‘readers’), and demethylases (‘erasers’) [6]. The prominent m^6^A methylation regulators consist of ‘writers’ such as methyltransferase like 3 (METTL3), METTL14, WT1-associated protein (WTAP), KIAA1429, RNA binding motif protein 15 (RBM15) and zinc finger CCCH domain-containing protein 13 (ZC3H13), ‘readers’ such as YTH domain-containing 1 (YTHDC1), YTH domain-containing 1 (YTHDC2), YTH N6-methyladenosine RNA binding protein 1 (YTHDF1), YTH N6-methyladenosine RNA binding protein 2 (YTHDF2) and heterogeneous nuclear ribonucleoprotein C (HNRNPC), and ‘erasers’ such as fat mass- and obesity-associated protein (FTO) and α-ketoglutarate-dependent dioxygenase alkB homolog 5 (ALKBH5) [6,16–21]. The discovery of m^6^A RNA methylation regulators has dramatically increased our understanding of the function and mechanism of m^6^A modification in the posttranscriptional regulation of gene expression [6,12,15,22,23].

m^6^A modification not only plays a vital role in oocyte and central nervous system development [9,16] but also has various regulatory functions in tumor initiation, progression and radio-resistance [4,22,24,25]. Moreover, increasing evidence indicates that genetic changes and dysregulated expression of m^6^A RNA methylation regulators are closely associated with malignant progression in various kinds of cancer [4,15,21,22,26,27]. Recently, an important study revealed that FTO expression is upregulated in *IDH*-mutant leukemia and plays an anti-tumor role through the FTO/m^6^A/MYC/CEBPA signaling pathway [28]. *IDH* mutation is also a major driver of mutation in glioma, and gliomas with *IDH* mutation have a better prognosis than *IDH* wildtype gliomas. The m^6^A RNA methylation regulators were also reported to play pivotal roles in glioma stem-like cell maintenance and radio-resistance [25,26]. However, the literature lacks a comprehensive analysis of the expression of m^6^A RNA methylation regulators in gliomas with different clinicopathological characteristics, their function in glioma malignant progression, and their prognostic value.

In this study, we systematically analyzed the expression of thirteen widely reported m^6^A RNA regulators in 904 gliomas with RNA sequencing data from the Chinese Glioma Genome Atlas (CGGA) (n = 309) and The Cancer Genome Atlas (TCGA) (n = 595) datasets. We provided the expression data of each m^6^A modification regulator with regard to different clinicopathological features. We found that the expression of m^6^A RNA methylation regulators plays important roles in the malignant progression of gliomas, and a signature with seven selected m^6^A RNA methylation regulators was designed to stratify the prognosis of gliomas.

## RESULTS

### Expression of m^6^A RNA methylation regulators is correlated with clinicopathological features in gliomas

**Figure 1:**
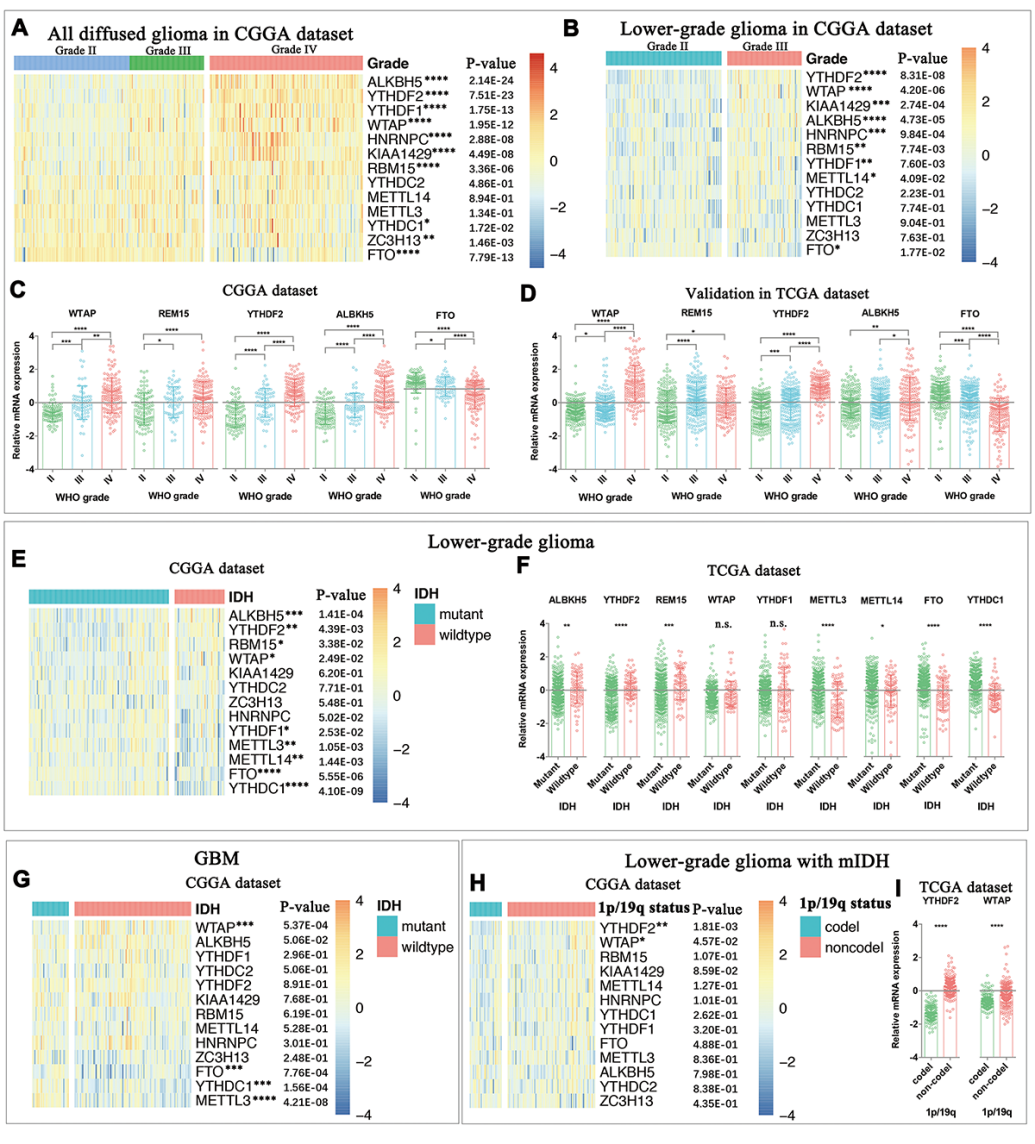
**Expression of m^6^A RNA methylation regulators in gliomas with different clinicopathological features.** (**A-D**) The expression levels of thirteen m^6^A RNA methylation regulators in gliomas with different WHO grades. (**E-F**) The expression levels of m^6^A RNA methylation regulators in LGG with different *IDH* status. (**G**) The expression levels of m^6^A RNA methylation regulators in GBM with different *IDH* status. (**H**) The expression levels of m^6^A RNA methylation regulators in *IDH*-mutant (m*IDH*) LGG with different 1p/19q codeletion status. * P < 0.05, ** P < 0.01, *** P < 0.001 and **** P < 0.0001.

Considering the important biological functions of each m^6^A RNA methylation regulator in tumorigenesis and development, we systematically investigated the relationships between each individual m^6^A RNA methylation regulator and the pathological features of gliomas, including WHO grade, *IDH* status, and 1p/19q codeletion status. The expression level of each m^6^A RNA methylation regulator and WHO grades are presented as heatmaps (Fig. 1A and B), showing that the expressions of most m^6^A RNA methylation regulators are significantly associated with WHO grades. The significant correlations between WHO grades and expression levels of WTAP, RBM15, YTHDF, ALBKH5, and FTO were also confirmed by quantitative analyses in both the CGGA (Fig. 1C) and TCGA datasets (Fig. 1D). As the WHO grade increased, the expression of WTAP, RBM15, YTHDF and ALBKH5 increased, while the expression of FTO decreased.

We then studied the relationship between *IDH* status and expression levels of each m^6^A RNA methylation regulator in lower-grade gliomas (LGG, Fig. 1E and 1F) and glioblastomas (GBM, Fig. 1G), respectively. The results showed that expression levels of ALKBH5, YTHDF2, RBM15, METTL3, METTL14, FTO and YTHDC1 are significantly different between LGG with mutant-*IDH* and LGG with wildtype-*IDH* in both CGGA (Fig. 1E) and TCGA (Fig. 1F) datasets. FTO, YTHDC1 and METTL3 were also differentially expressed between GBM with and without *IDH* mutation in the CGGA dataset (Fig. 1G). However, we were unable to compare the expression of m^6^A RNA methylation regulators between GBM with and without *IDH* mutation in the TCGA dataset because of the dramatically imbalanced numbers of *IDH*-mutant GBM (n=10) and *IDH*-wildtype GBM (139). In the LGG with mutant-*IDH*, we observed that the expressions of YTHDF2 and WTAP were highly correlated with the status of 1p/19q codeletion (1p/19q codel).

We noticed that the expression of both METTL3 and METTL14 were not correlated with the WHO grade of gliomas. Considering METTL16 is also a putative m^6^A methyltransferase and knockdown of METTL16 could result in approximately 20% decrease of m^6^A [29], we also investigated the expression of METTL16 in gliomas with different malignancy status in the CGGA dataset (Figure S1). METTL16 expression was also not correlated with the WHO grade of gliomas. Though METTL16 expression correlated to the *IDH* status in LGG (P<0.001) and GBM (P<0.05), it did not have prognostic value in any pathological subgroup of gliomas (data not shown). In addition, the expression information was also not included in the TCGA dataset, so METTL16 was not included in the following section of this study.

Moreover, we also observed that the genetic change (mutation or coy number change) frequencies of the thirteen m^6^A RNA methylation regulators were very low (all ≤ 1.1%) in gliomas (Figure S2), indicating that the expression changes of these m^6^A RNA methylation regulators were not caused by the genetic changes of the corresponding genes.

### Consensus clustering of m^6^A RNA methylation regulators identified two clusters of gliomas with distinct clinical outcomes and clinicopathological features

**Figure 2:**
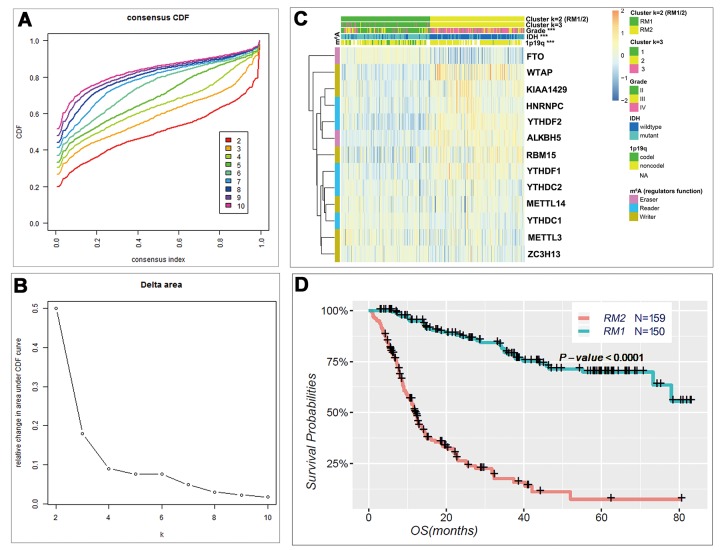
**Differential clinicopathological features and overall survival of gliomas in the RM1/2 subgroups.** (**A**) Consensus clustering cumulative distribution function (CDF) for k = 2 to 10. (**B**) Relative change in area under CDF curve for k = 2 to 10. (**C**) Heatmap and clinicopathologic features of the two clusters (RM1/2) defined by the m^6^A RNA methylation regulators consensus expression. (**D**) Kaplan–Meier overall survival (OS) curves for 309 CGGA glioma patients.

Based on the expression similarity of m^6^A RNA methylation regulators, k = 3 seemed to be an adequate selection with clustering stability increasing from k = 2 to 10 in the CGGA datasets (Fig. 2A and B). However, we noticed that 293 out of 309 gliomas clustered into one of the two subgroups in the CGGA dataset (Fig. 2C and Figure S3). Thus, we compared the clinicopathological features of these two subgroups clustered by k=2, namely, RM1 and RM2 (Fig. 2C) datasets. The RM1 subgroup is significantly correlated with younger age at diagnosis (P < 0.0001), lower grade (P < 0.0001), proneural or neural subtypes (P < 0.0001), *IDH*-mutational status (P < 0.0001) and 1p/19q codel status (P < 0.0001). The RM2 subgroup mainly contains gliomas with an older age at diagnosis, glioblastoma (GBM) phenotype, classic or mesenchymal subtypes, *IDH*-wildtype status and 1p/19q noncodel status (Table S2). Moreover, we observed a significantly shorter overall survival (OS) in the RM2 subgroup than the RM1 subgroup (Fig. 2D).

### Categories identified by consensus clustering are closely correlated to the malignancy of gliomas

**Figure 3:**
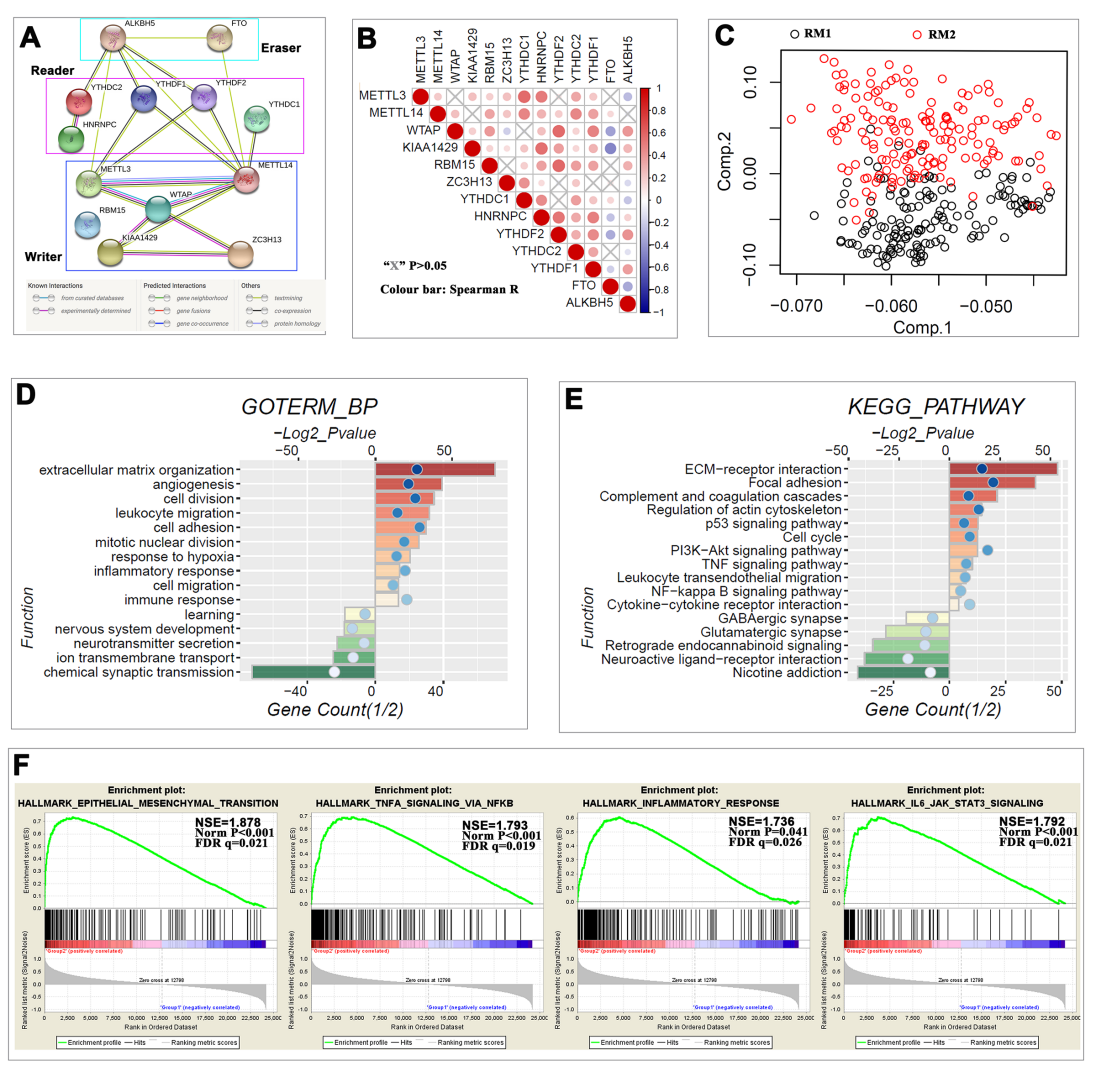
**Interaction among m^6^A RNA methylation regulators and functional annotation of gliomas in RM1/2 subgroups.** (**A**) The m^6^A modification-related interactions among the 13 m^6^A RNA methylation regulators. (**B**) Spearman correlation analysis of the 13 m^6^A modification regulators. (**C**) Principal component analysis of the total RNA expression profile in the CGGA dataset. Gliomas in the RM2 subgroup are marked with red. (**D–E**) Functional annotation of the genes with higher expression in the RM2 subgroup (red bar chart) or RM1 subgroup (green bar chart) using GO terms of biological processes (**D**) and KEGG pathway (**E**). (**F**) GSEA revealed that genes with higher expression in RM2 subgroup were enriched for hallmarks of malignant tumors.

The above findings suggested that the clustering result was closely correlated to the malignancy of the gliomas. To better understand the interactions among the thirteen m^6^A RNA methylation regulators, we also analyzed the interaction (Fig. 3A) and correlation (Fig. 3B) among these regulators. WTAP seems to be the hub gene of the ‘writers’, and its interactions or coexpressions with METTL3, METTL14, KIAA1429 and ZC3H13 are supported both by experimental data and by text mining in the String database (Fig. 3A). The expression of WTAP was also significantly correlated with the ‘writers’ of METTL14, KIAA1429 and RBM15 in gliomas (Fig. 3B). There are several independent interaction groups in ‘readers’, suggesting the diverse functions of different ‘readers’ (Fig. 3A), but the expressions of YTHDF2, YTHDC2 and YTHDF1 were significantly correlated with each other in gliomas (Fig. 3B). Only text mining evidence supported the interaction of ALKBH5 and FTO in the String database (Fig. 3A), and the expressions of ALKBH5 and FTO were negatively correlated with each other in gliomas (Fig. 3B). Moreover, the expressions of WTAP, RBM15, YTHDF2, YTHDF1 and ALKBH5 were highly correlated with each other, and all of their expressions were negatively correlated with FTO in gliomas (Fig. 3B). These findings were consistent with that the expression levels of WTAP, RBM15, YTHDF2, YTHDF1 and ALKBH5 being positively correlated with the increasing malignancy of gliomas, while the expression levels of FTO are negatively correlated with the increasing malignancy of gliomas (Figs. 1 and 2).

We further used principal component analysis (PCA) to compare the transcriptional profile between RM1 and RM2 subgroups. The results showed a clear distinction between them (Fig. 3C). We identified genes that were significantly upregulated (Score(d) for SAM > 8, fold change >2, and normalized P < 0.01) or downregulated (Score(d) for SAM < -8, fold change < 0.5, and normalized P < 0.01) in the RM2 subgroup, and then annotated their function using gene ontology (GO) pathway analysis for biological processes (Fig. 3D). The results indicated that upregulated genes are enriched in malignancy-related biological processes, including cell proliferation, extracellular matrix organization, angiogenesis, migration, and immune response, among others. Similar changes in corresponding signaling pathways were also observed in the Kyoto Encyclopedia of Genes and Genomes (KEGG) pathway analysis (Fig. 3E). Furthermore, gene set enrichment analysis (GSEA) revealed that the malignant hallmarks of tumors, including epithelial-mesenchymal transition (NES=1.88, normalized P < 0.001), TNFα signaling via NF-κB (NES=1.79, normalized P < 0.001), inflammatory response (NES=1.74, normalized P = 0.041) and IL6/JAK/ STAT3 signaling (NES=1.79, normalized P < 0.001), were significantly associated with the RM2 subgroup (Fig. 3F). All of these findings indicated that the two categories identified by consensus clustering are closely correlated with the malignancy of gliomas.

### Prognostic value of m^6^A RNA methylation regulators, and a risk signature built using seven selected m^6^A RNA methylation regulators

**Figure 4:**
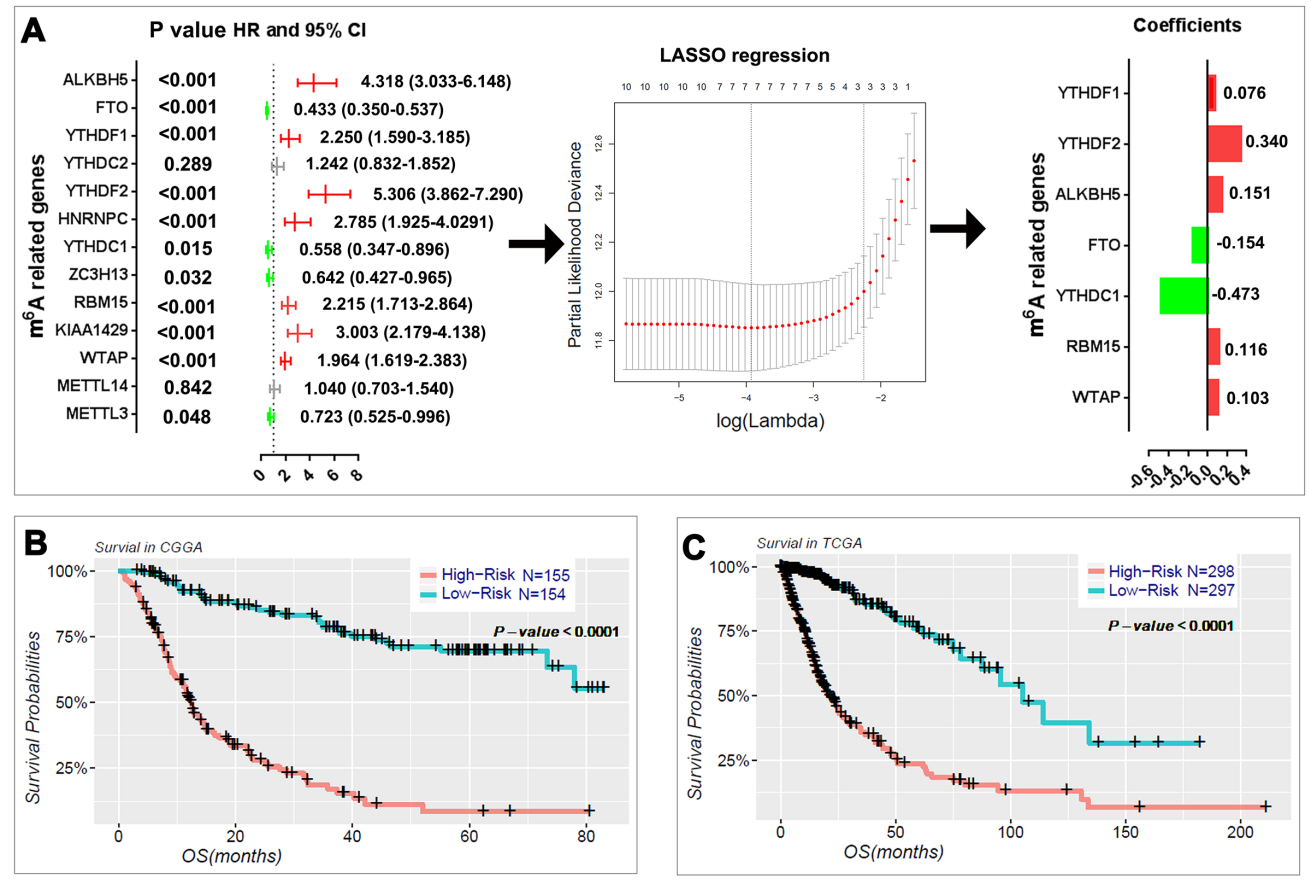
**Risk signature with seven m^6^A RNA methylation regulators.** (**A**) The process of building the signature containing seven m^6^A RNA methylation regulators. The hazard ratios (HR), 95% confidence intervals (CI) calculated by univariate Cox regression and the coefficients calculated by multivariate Cox regression using LASSO are shown. (**B–C**) Kaplan–Meier overall survival (OS) curves for patients in the CGGA (**B**) and TCGA (**C**) datasets assigned to high- and low-risk groups based on the risk score.

We next sought to investigate the prognostic role of m^6^A RNA methylation regulators in gliomas. We performed a univariate Cox regression analysis on the expression levels in the CGGA dataset (Fig. 4A). The results indicated that eleven out of thirteen tested genes are significantly correlated with OS (P < 0.05). Among these eleven genes, ALKBH5, YTHDF1, YTHDF2, HNRNPC, RBM15, KIAA1429, and WTAP are risky genes with HR > 1, while FTO, YTHDC1, ZC3H13, and METTL3 are protective genes with HR < 1. We also investigated the prognostic value for each m^6^A RNA methylation regulator in stratified LGG (Table S3) and GBM (Table S4). For the genes with prognostic value in LGG, the expression levels of YTHDF2, WTAP, ALKBH5, RBM15, KIAA1429, HNRNPC, YTHDF1, and FTO are significantly correlated with the overall survival (OS) of patients with *IDH*-mutant and 1p/19q noncodel LGG. Among these genes, YTHDF2, KIAA1429, HNRNPC, and YTHDF1 also have prognostic value in *IDH*-wildtype LGG. In the GBM, the expressions of FTO, YTHDF2, and RBM15 have prognostic value in *IDH*-wildtype GBM; and FTO also has prognostic value in *IDH*-mutant GBM.

To better predict the clinical outcomes of gliomas with m6A RNA methylation regulators, we applied the least absolute shrinkage and selection operator (LASSO) Cox regression algorithm to the eleven prognosis-associated genes in the CGGA dataset, which we used as a training set (Fig. 4A). Seven genes were selected to build the risk signature based on the minimum criteria, and the coefficients obtained from the LASSO algorithm were used to calculate the risk score for both the training dataset (CGGA) and the validation dataset (TCGA). To investigate the prognostic role of the seven-gene risk signature, we separated the glioma patients in the CGGA (n = 309) and TCGA (n = 595) datasets into low- and high-risk groups based on the median risk score and observed significant differences in OS between the two categories (both P < 0.0001; Fig. 4B, C).

### Prognostic risk scores showed strong associations with clinicopathological features in gliomas

**Figure 5:**
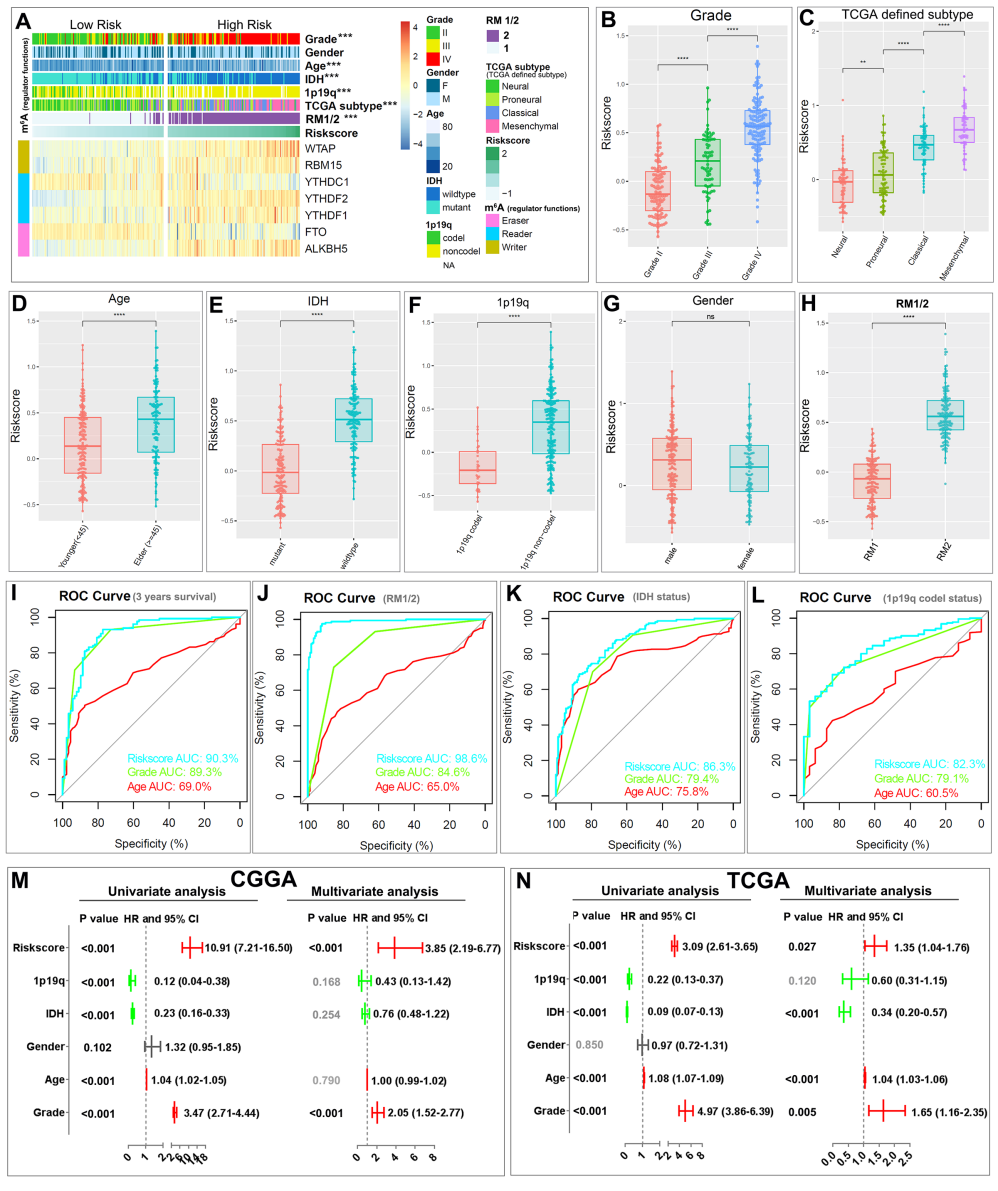
**Relationship between the risk score, clinicopathological features and RM1/2 subgroups.** (**A**) The heatmap shows the expression levels of the seven m^6^A RNA methylation regulators in low- and high-risk gliomas. The distribution of clinicopathological features was compared between the low- and high-risk groups. *** P<0.001 (**B–H**) Distribution of risk scores in the CGGA dataset stratified by WHO grade (**B**), TCGA subtype (**C**) age (**D**), *IDH* status (**E**), 1p/19q codel status (**F**), gender (**G**) and RM1/2 subgroups (**H**). **P < 0.01, and ****P < 0.0001. (**I-L**) ROC curves showed the predictive efficiency of the risk signature, WHO grade, and age on the three-year survival rate (**I**), RM1/2 subgroups (**J**), *IDH*-mutant status (**K**) and 1p/19q codel status (**L**). (**M-N**) Univariate and multivariate Cox regression analyses of the association between clinicopathological factors (including the risk score) and overall survival of patients in the CGGA (**M**) and TCGA (**N**) datasets. ns no significance, *** P < 0.001 and **** P < 0.0001.

The heatmap shows the expression of the seven selected m^6^A RNA methylation regulators in high- and low-risk patients in the CGGA dataset (Fig. 5A). We observed significant differences between the high- and low-risk groups with respect to WHO grade (P < 0.001), age (P < 0.001), *IDH* status (P < 0.001), 1p/19q codel status (P < 0.001), TCGA subtypes (P < 0.001), and RM1/2 subgroups (P < 0.001). We also examined the association between the risk scores and each clinicopathological feature. We observed that risk scores are significantly different between patients stratified by WHO grade, TCGA subtype, age, *IDH* status, 1p/19q codel status, and RM1/2 subgroups, but not by gender, in both the CGGA (Fig. 5B–H) and TCGA datasets (Figure S4). The ROC curve shows that the risk score can perfectly predict three-year survival rates for glioma patients (AUC = 90.3%), RM1/2 subgroups (AUC = 98.6%), glioma *IDH*-mutant status (AUC = 86.3%) and glioma 1p/19q codel status (AUC = 82.3%). Furthermore, the predictive efficiency is better than that of the WHO grade and age (Fig. 5I-L). These results indicate that the risk scores calculated with the signature can accurately predict glioma patient outcomes and clinicopathological features, especially for the RM1/2 subgroups.

We then performed univariate and multivariate Cox regression analyses for the CGGA dataset to determine whether the risk signature is an independent prognostic indicator. By univariate analysis, the risk score, 1p/19q codel status, *IDH* status, age and WHO grade were all correlated with the OS. When including these factors into the multivariate Cox regression, the risk score and WHO grade remained significantly associated with the OS (both P < 0.001, Fig. 5M). Similar results were found in the validation of the TCGA dataset; the risk score (P = 0.027), *IDH* status (P < 0.001), age (P < 0.001) and WHO grade (P = 0.005) remained significantly associated with the OS in the multivariate regression (Fig. 5N). These results confirmed that the risk score derived from m^6^A RNA methylation regulators can independently predict prognosis in glioma patients.

**Figure 6:**
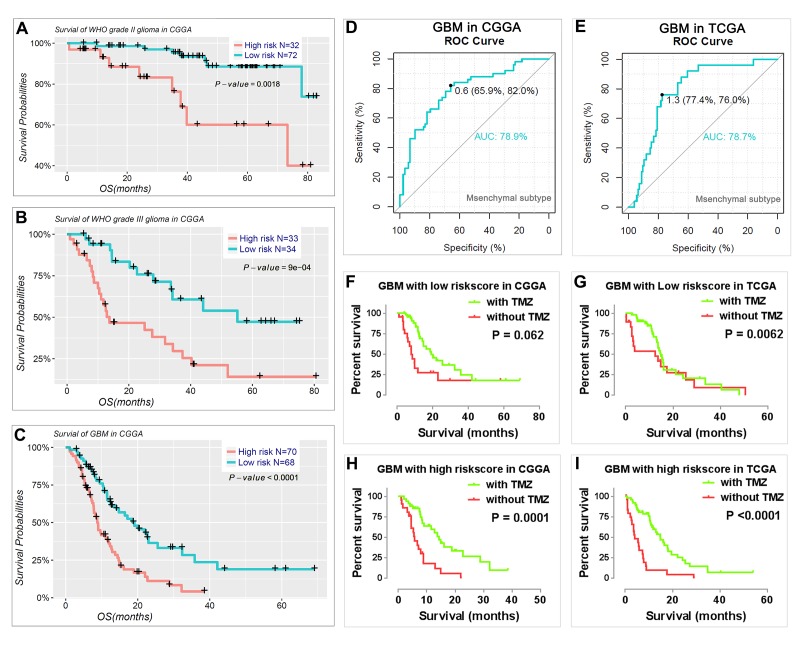
**Prognostic value of the risk signature in patients stratified by WHO grade.** (**A–C**) Kaplan–Meier overall survival curves for patients with WHO grade II (**A**), WHO grade III (**B**), and GBM (**C**). (**D-E**) ROC curves showed the predictive efficiency of the risk signature on mesenchymal subtype in GBM of CGGA (**D**) and TCGA (**E**) datasets. (**F-I**) GBM patients with high risk scores had a greater benefit from TMZ chemotherapy.

We also determined the prognostic value of the risk signature for different WHO grades. We found that patients with high risk scores had significantly shorter OS than those with low scores in WHO grade II and III gliomas (Fig. 6A, B) and GBM (Fig. 6C). Meanwhile, we also observed that the risk score had prognostic value in gliomas stratified by the integrated diagnosis of WHO grade 2016 (Figure S5). Moreover, the risk score could also predict the mesenchymal subtype in GBM for both CGGA (Fig. 6D) and TCGA datasets (Fig. 6E). Consistent with this finding, the patients with a high-risk score were also more sensitive to TMZ treatment in GBM for both CGGA and TCGA datasets (Fig. 6F-I).

## DISCUSSION

Malignant progression and high recurrence rate render gliomas the most lethal type of primary brain tumor [30–32]. Traditional epigenetics, limited to DNA or protein modification, has a variety of functions in glioma initiation, malignant progression, and prognosis [33–35]. In this study, we demonstrated that the expression of regulators of another area of epigenetics—RNA m^6^A RNA methylation—is also closely associated with the malignancy and prognosis of gliomas. We identified two glioma subgroups, RM1/2, by consensus clustering based on the expression of m^6^A RNA methylation regulators. The RM1/2 subgroups not only influenced the prognosis and clinicopathological features but were also closely correlated with biological processes, key signaling pathways, and hallmarks of malignant gliomas. In addition, we also derived a prognostic risk signature with seven selected m^6^A RNA methylation regulators, which stratified the OS of patients with gliomas into high- and low-risk categories.

Among the m^6^A RNA methylation regulators, the writers METTL3 and METTL14 were reported as suppressor genes in GBM but oncogenes in acute myelocytic leukemia (AML) and hepatocellular carcinoma [15,26]; the reader YTHDF2 promotes cancer cell proliferation in pancreatic cancer [36]; the eraser ALKBH5 functions as an oncogene in both GBM and breast cancer [4,37]; and the eraser FTO is an oncogene in AML and glioma [4,28]. These findings suggested that up- or downregulation of specific RNA m^6^A methylation regulators are linked to mis-regulated RNAs in tumors, and the same m^6^A methylation regulators may have distinct functions in different tumors.

In this section, we comprehensively analyzed the expression of all m^6^A RNA methylation regulators in gliomas with different clinicopathological features. As an m^6^A methylation writer, the expression of METLL3 was decreased in the mesenchymal subtype or *IDH*-wildtype gliomas. WTAP expression was significantly increased in high-grade, mesenchymal subtype, *IDH*-wildtype, 1p/19q noncodel and elderly glioma patients, indicating potential functions of WTAP in glioma malignancy. For the m^6^A methylation readers, the expression of HNRNPC, YTHDF1, and YTHDF2 was significantly increased in high-grade gliomas. Furthermore, the expression of YTHDF2 is highly correlated with 1p/19q codeletion (1p/19q codel) status. For m^6^A methylation erasers, although both ALKBH5 and FTO were reported as oncogenes in GBM [4], their expression varies dramatically across different clinicopathological features. Interestingly, the expression of FTO was significantly deceased in gliomas with malignant clinicopathological features, such as higher WHO grade, mesenchymal subtype, *IDH*-wildtype status, 1p/19q noncodel and older ages at diagnosis. Unlike ALKBH5, FTO could mediate the demethylation of both internal m^6^A and N6, 2-O-dimethyladenosine (m^6^Am) at the +1 position from the 5' cap in mRNA [38]. Recently, FTO was found to preferentially mediate the demethylation of m^6^Am rather than of m^6^A [39]. This also indicates that FTO and ALKBH5 may preferentially mediate demethylation of different methylation targets in glioma, and it is worth investigating in future studies. Taken together, the expression of m^6^A RNA methylation regulators is closely associated with malignant clinicopathological features in gliomas. Moreover, these findings are also useful for developing novel therapeutic methods through characterizing the expression of each individual m^6^A methylation regulator in gliomas, as chemicals targeting m^6^A methylation are considered a new method for cancer therapy [22,40].

This study uncovered that the m^6^A RNA methylation regulators were also correlated with the biological processes and signaling pathways of glioma malignant progression. The role of RNA m^6^A methylation in cancer has only been ascertained in recent years. Several biological processes and signaling pathways that it affects have been identified, including tumor stem cell growth; self-renewal, and tumorigenesis [4,26]; RNA metabolism, including mRNA/miRNA/ lncRNA biogenesis, processing and exporting [22]; DNA damage response secondary to radio- or chemotherapy [22,41]; the IL-7/STAT5/SOCS pathways [42]; and the FTO/m^6^A/MYC/enhancer-binding protein alpha signaling pathway [28]. Here, we revealed that in glioma, the expression of m^6^A RNA methylation regulators is significantly associated with biological processes, such as extracellular matrix, angiogenesis, cell division, response to hypoxia, inflammatory response, immune response and others, as well as with signaling pathways, such as ECM-receptor interaction, p53, cell cycle, PI3K-Akt, TNF, NFκB and others.

Whether the expression level of m^6^A RNA methylation regulators can be used as a prognostic maker is an important topic of research [4]. In this study, our glioma prognostic signature derived using seven m^6^A RNA methylation regulators was found to be of value. In addition, we also determined the prognostic value of the signature for WHO grades II, III, and IV gliomas in both the CGGA and TCGA dataset. Moreover, the risk score also had prognostic value in gliomas stratified by WHO 2016 grades (Figure S5). As we observed, though the signature risk score can stratify the OS for *IDH*-mutant and 1p/19q noncodel lower grade gliomas, *IDH*-wildtype lower grade gliomas, and *IDH*-wildtype GBM in the CGGA dataset, it cannot distinguish the OS in the TCGA dataset (data not shown). A similar scenario was also observed in the multivariate COX analysis. This may be caused by the strong correlation between the risk signature and *IDH* status.

**Figure 7:**
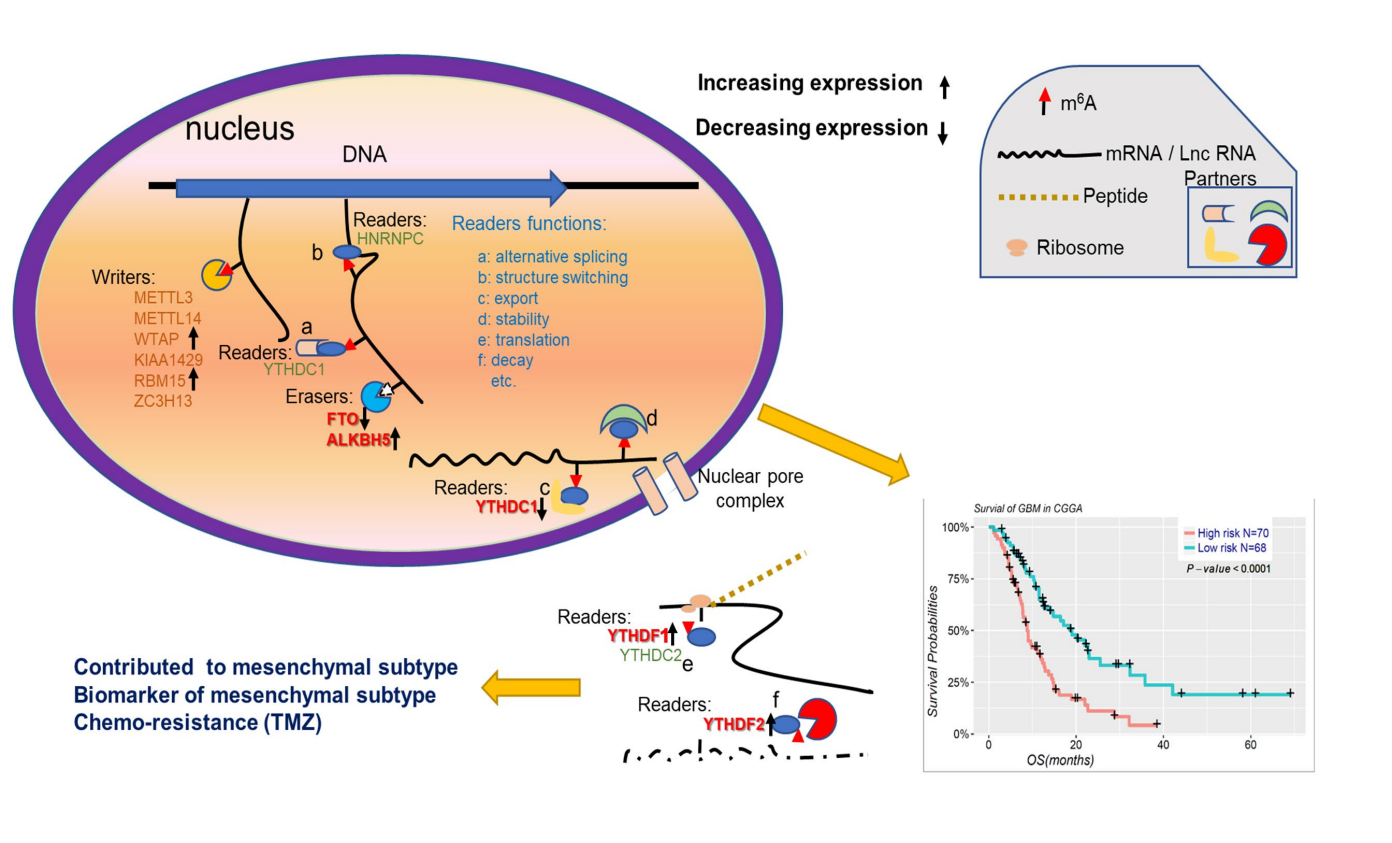
Summary for the expression changes and potential functions of m^6^A RNA methylation regulators in gliomas.

In conclusion, our findings systematically demonstrated the expression, potential function, and prognostic value of m^6^A RNA methylation regulators in gliomas (Figure 7). The expressions of m^6^A RNA methylation regulators, which are highly associated with the malignant clinicopathological features of gliomas, are also significantly correlated with the increased expression levels of genes enriched in the biological processes and signaling pathways that promote the malignant progression of gliomas. In summary, our study provides important evidence for future examination of the role of RNA m^6^A methylation in gliomas.

## MATERIALS AND METHODS

### Datasets

The RNA-seq transcriptome data and corresponding clinicopathological information were obtained for 309 glioma patients from CGGA (www.cgga.org.cn) and 595 glioma patients from TCGA (http://cancergenome.nih.gov/). For the RNA-seq data, reads per kilobase of transcript per million mapped reads values were obtained for the CGGA samples [27], and values normalized by expectation-maximization were obtained for the TCGA samples. The study protocol was approved by the ethics committee of Beijing Tiantan Hospital. Clinicopathological information for the CGGA and TCGA datasets is summarized in Supplementary Table S1.

### Selection of m^6^A RNA methylation regulators

We first collated a list of sixteen m^6^A RNA methylation regulators from published literature [6,15,21], and then we restricted the list to genes with available RNA expression data in the CGGA and TCGA datasets. This yielded a total of thirteen m^6^A RNA methylation regulators. Then, we systematically compared the expression of these m^6^A RNA methylation regulators in gliomas with different clinicopathological features.

### Bioinformatic analysis

To investigate the function of m^6^A RNA methylation regulators in gliomas, we clustered the gliomas into different groups with “ConsensusClusterPlus” (50 iterations, resample rate of 80%, and Pearson correlation, http://www.bioconductor.org/). We used PCA with the R package for R v3.4.1 to study the gene expression patterns in different glioma groups. GO and KEGG pathway enrichment analyses were performed with the Database for Annotation, Visualization, and Integrated Discovery (http://david.abcc.ncifcrf.gov/home.jsp) to functionally annotate genes that are differentially expressed in different groups. Interactions among m^6^A RNA methylation regulators were analyzed using the STRING database (http://www.string-db.org/). GSEA was performed to investigate the functions correlated with different subgroups of gliomas.

To determine the prognostic value of m^6^A RNA methylation regulators, we performed univariate Cox regression analyses of their expression in the CGGA dataset. From this, we identified eleven genes significantly associated with survival (P < 0.05), which we selected for further functional analysis and development of a potential risk signature with the LASSO Cox regression algorithm [43–46]. Finally, seven genes and their coefficients were determined by the minimum criteria, selecting the best penalty parameter λ associated with the smallest 10-fold cross validation within the training set. The risk score for the signature was calculated using the formula:

$$\text{Risk score = }\sum_{i=1}^{n}{\text{Coef}}_{\text{i}}\times x_{\text{i}}$$,

where Coef_i_ is the coefficient, and $x_{i}$ is the z-score-transformed relative expression value of each selected gene. This formula was used to calculate a risk score for each patient in both the training (CGGA) and validation (TCGA) datasets.

### Statistical analysis

One-way ANOVA was used to compare the expression level of m^6^A RNA methylation regulators in gliomas with different WHO grades and TCGA subtypes, and t-tests were used to compare the expression levels in gliomas for age, gender, *IDH* status, and 1p/19q codel status.

Patients were clustered into two groups by consensus expression of m^6^A RNA methylation regulators or were divided into high- and low-risk groups using the median risk score (derived from the risk signature) as the cutoff value. Chi-square tests were used to compare the distribution of gender, WHO grade, TCGA subtype, *IDH* status, and 1p/19q codeletion status between the two risk groups.

To compare the risk scores of the signature for gliomas with different clinicopathologies, a one-way ANOVA or t-test was performed to compare the risk scores in patients grouped by clinical or molecular-pathological characteristics. Univariate and multivariate Cox regression analyses were performed to determine the prognostic value of the risk score and various clinical and molecular-pathological characteristics.

The prediction efficiency of the risk signature, WHO grade and age for 3-year survival, RM1/2 groups, *IDH*-mutant status and 1p/19q codeletion status were tested with operating characteristic (ROC) curves.

The Kaplan–Meier method with a two-sided log-rank test was used to compare the OS of the patients in the RM1/2 groups or in the high- and low-risk groups. All statistical analyses were conducted using R v3.4.1 (https://www.r-project.org/), SPSS 16.0 (SPSS Inc., Chicago, IL) and Prism 7 (GraphPad Software Inc., La Jolla, CA).

## SUPPLEMENTARY MATERIALS

Supplementary Figures

Supplementary Tables
