# Supplementary material for: m6A RNA methylation regulators contribute to malignant progression and have clinical prognostic impact in gliomas
Source: Aging (Albany NY). 2019 Feb 27;11(4):1204–25. doi: 10.18632/aging.101829 (PMC6402513; doi:10.18632/aging.101829)
Supplement: Supplementary Figures [file aging-11-101829-s001.pdf]

SUPPLEMENTARY MATERIAL

Supplementary Figures

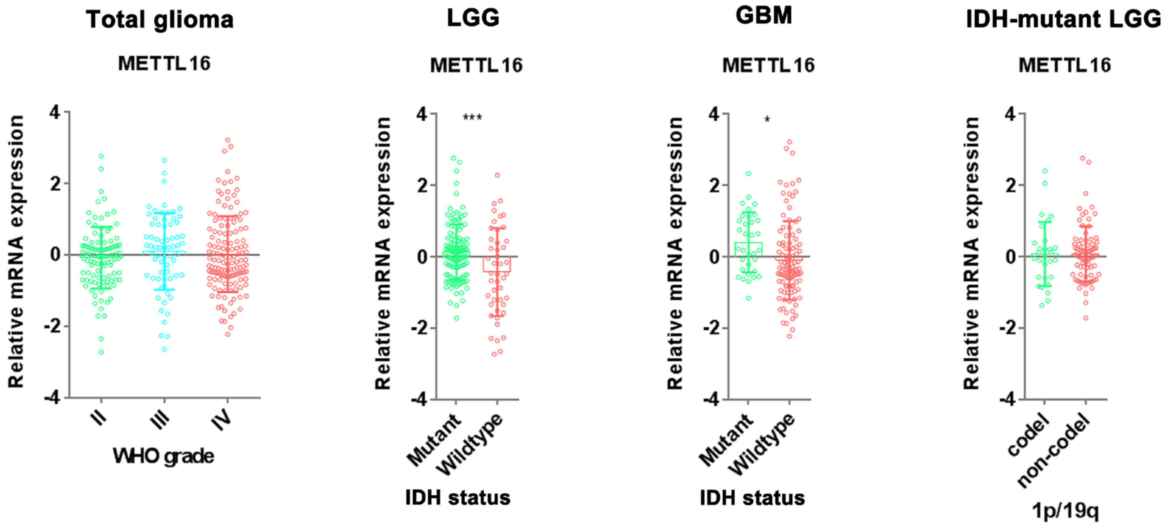

Figure S1. Expression of METTL16 in gliomas with different clinicopathological features.

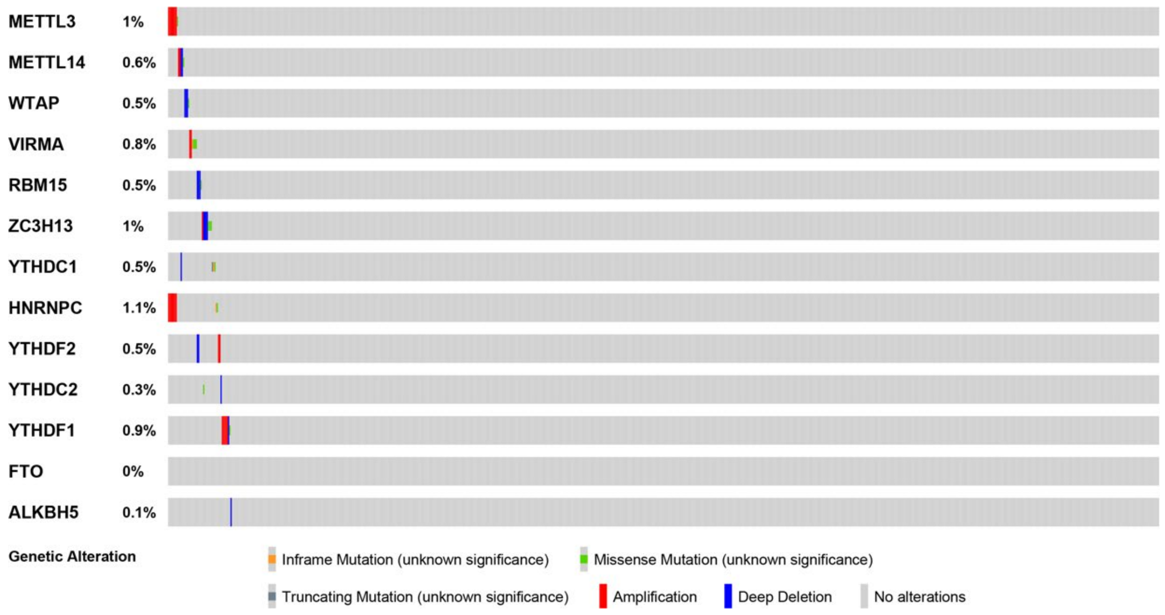

Figure S2. Genetic changes of RNA m[6]A regulators in TCGA dataset. Genetic changes of the thirteen m[6]A regulators in the 595 gliomas from the TCGA dataset.

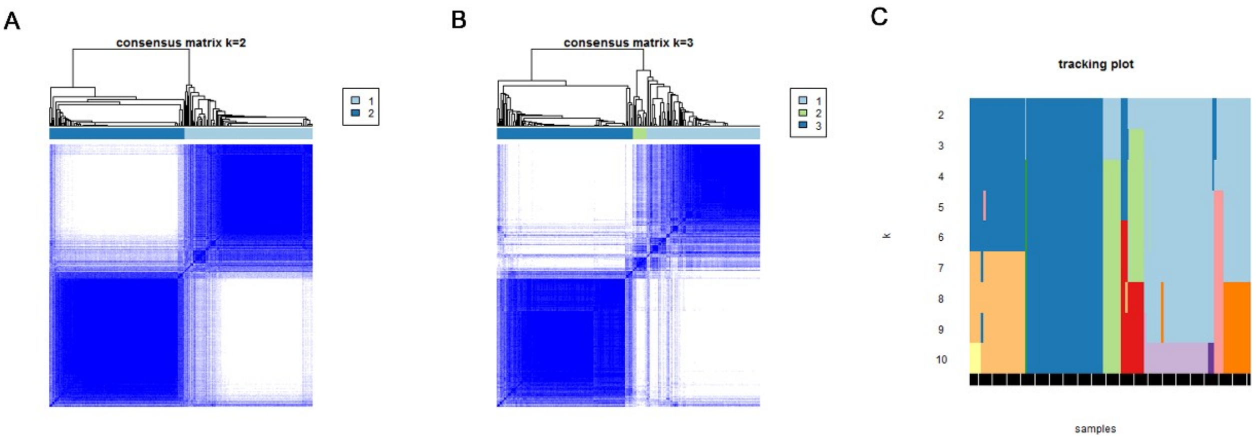

Figure S3. Identification of consensus clusters by m^{6}A RNA methylation regulators. (**A-B**) Consensus clustering matrix for k = 2 (**A**) and k = 3 (**B**). (C) the tracking plot for k=2 to k=10.

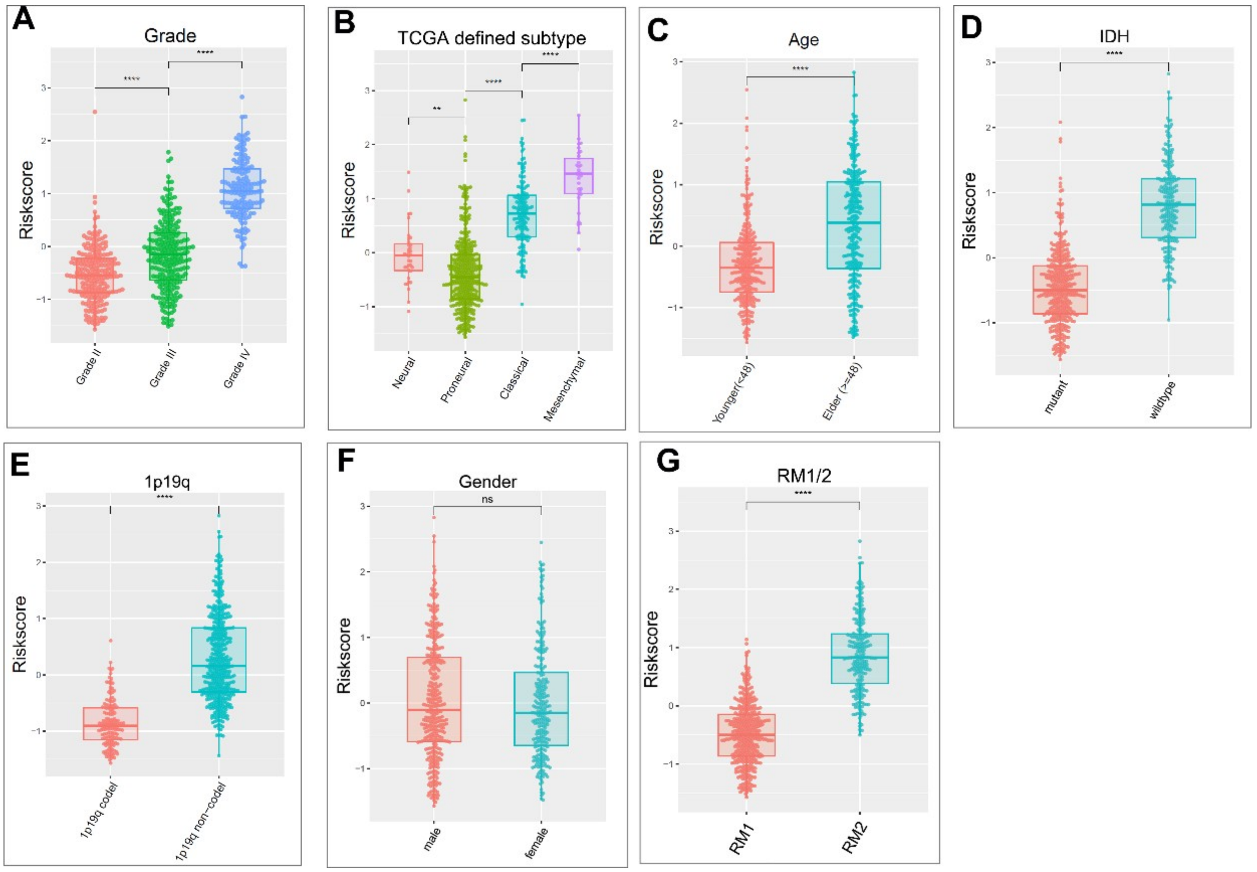

Figure S4. Relationship between the risk score, clinicopathological features and RM1/2 subgroups in the TCGA dataset. (**A–G**) Distribution of risk scores in the TCGA dataset stratified by WHO grade (A), TCGA subtype (B), age (C), *IDH* status (D), 1p/19q codel status (E), gender (F) and RM1/2 subgroups(G). ns no significance, ** $P < 0.01$, and **** $P < 0.0001$.

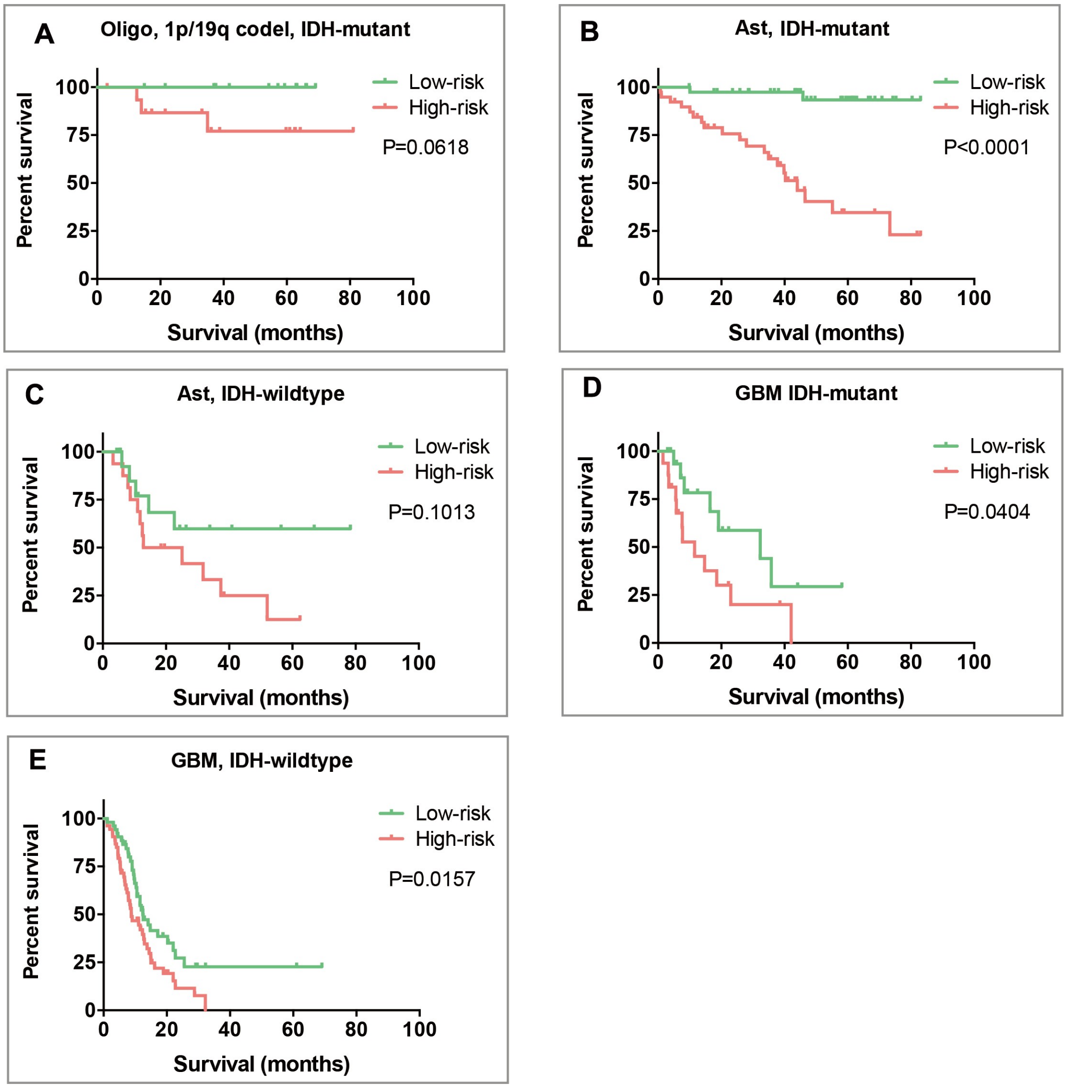

Figure S5. Prognostic value of the risk signature in patients stratified by the integrated analysis of WHO 2016. (**A-E**) Kaplan–Meier overall survival curves for patients with Oligodendroglioma with IDH-mutant and 1p/19q co-deletion (**A**), Astrocytoma with IDH-mutant (**B**), Astrocytoma with IDH-wildtype (**C**), GBM with IDH-mutant (**D**), and GBM with IDH- wildtype (**E**).
