# Supplementary material for: m6A RNA methylation regulators contribute to malignant progression and have clinical prognostic impact in gliomas
Source: Aging (Albany NY). 2019 Feb 27;11(4):1204–25. doi: 10.18632/aging.101829 (PMC6402513; doi:10.18632/aging.101829)
Supplement: Supplementary Tables [file aging-11-101829-s002.pdf]

Supplementary Tables

Table S1. Clinicopathological features of patients included in this study.

		CGGA dataset		TCGA dataset	
		Number	Percentage	Number	Percentage
**Total**		309	100.00%	595	100.00%
**Age**		8-81 (43)		14-89 (47)	
	< median	153	49.51%	292	49.08%
	≥ median	156	50.49%	303	50.92%
**Gender**					
	Female	115	37.22%	248	41.68%
	Male	194	62.78%	343	57.65%
**Grade**			0.00%		0.00%
	II	104	33.66%	211	35.46%
	III	67	21.68%	235	39.50%
	IV	138	44.66%	149	25.04%
**IDH**					
	Mutation	159	51.46%	373	62.69%
	Wildtype	150	48.54%	222	37.31%
**1p19q**					
	Codel	31	10.03%	148	24.87%
	Non-codel	220	71.20%	447	75.13%
	NA	58	18.77%	0	0.00%

Table S2. Clinicolpathological features are different between RM1 and RM2.

		RM1	RM2	P-value
Total cases		150	159	
Gender				6.739E-02
	Male	89	105	
	Female	61	54	
Age		40 (10-75)	46 (8-81)	2.460E-05
Grade				4.593E-150
	II	93	11	
	III	35	32	
	IV	22	116	
TCGA subtype				2.593E-108
	Neural	67	9	
	Proneural	67	32	
	Classical	13	56	
	Mesenchymal	3	62	
IDH				3.605E-60
	Mutant	122	37	
	Wildtype	28	122	
1p19q				1.137E-81
	Codel	29	2	
	Non-codel	96	124	
	NA	25	33	

Table S3. The prognosis value of m6A regulators in LGG.

m6A regulators	LGG				LGG, IDH-wildtype				LGG, IDH-mutant, 1p/19q noncodel				LGG, IDH-mutant, 1p/19q codel			
	HR	Confident Interval		P-value	HR	Confident Interval		P-value	HR	Confident Interval		P-value	HR	Confident Interval		P-value
		low	high			low	high			low	high			low	high	
**YTHDF2**	**2.76**	**2.08**	**3.66**	**1.79E-12**	**1.79**	**1.04**	**3.09**	**3.69E-02**	**6.78**	**3.25**	**14.12**	**3.24E-07**	18.65	0.56	625.01	1.03E-01
**WTAP**	**1.86**	**1.43**	**2.41**	**3.67E-06**	1.27	0.90	1.80	1.77E-01	**2.04**	**1.31**	**3.17**	**1.58E-03**	263.02	0.38	180022.12	9.44E-02
**ALKBH5**	**2.24**	**1.59**	**3.18**	**4.89E-06**	1.35	0.79	2.32	2.77E-01	**3.10**	**1.57**	**6.13**	**1.12E-03**	10.06	0.12	831.49	3.06E-01
**RBM15**	**1.86**	**1.38**	**2.49**	**4.06E-05**	1.23	0.75	2.02	4.02E-01	**2.21**	**1.34**	**3.65**	**1.85E-03**	1.89	0.27	13.44	5.24E-01
**KIAA1429**	**1.96**	**1.41**	**2.73**	**7.24E-05**	**1.85**	**1.10**	**3.11**	**2.11E-02**	**2.19**	**1.31**	**3.66**	**2.69E-03**	2.51	0.07	86.05	6.10E-01
**HNRNPC**	**2.05**	**1.43**	**2.95**	**9.67E-05**	**1.52**	**1.03**	**2.24**	**3.29E-02**	**3.78**	**1.79**	**7.98**	**5.02E-04**	6.23	0.13	295.15	3.53E-01
**YTHDF1**	**1.61**	**1.15**	**2.26**	**5.56E-03**	**1.79**	**1.04**	**3.09**	**3.69E-02**	**2.56**	**1.28**	**5.15**	**8.19E-03**	18.58	0.05	7576.31	3.41E-01
**FTO**	**0.64**	**0.47**	**0.88**	**5.92E-03**	0.98	0.61	1.59	9.38E-01	**0.28**	**0.10**	**0.75**	**1.15E-02**	0.06	0.00	8.77	2.68E-01
METTL14	1.28	0.94	1.75	1.20E-01	1.37	0.94	1.99	1.00E-01	1.27	0.77	2.11	3.47E-01	3.18	0.01	707.04	6.74E-01
ZC3H13	0.85	0.61	1.18	3.24E-01	1.15	0.71	1.88	5.65E-01	**0.60**	**0.39**	**0.94**	**2.47E-02**	0.57	0.00	91.98	8.27E-01
YTHDC2	1.05	0.78	1.43	7.36E-01	1.21	0.78	1.87	3.91E-01	0.86	0.52	1.42	5.57E-01	0.63	0.03	14.74	7.73E-01
METTL3	1.00	0.74	1.37	9.80E-01	1.11	0.78	1.58	5.70E-01	0.92	0.53	1.57	7.49E-01	6.50	0.13	322.61	3.47E-01
YTHDC1	1.00	0.73	1.38	9.86E-01	1.35	0.88	2.06	1.67E-01	1.17	0.65	2.11	6.09E-01	0.64	0.00	229.68	8.83E-01

Table S4. The prognosis value of m6A regulators in GBM.

m6A regulators	GBM				GBM, IDH-wildtype				GBM, IDH-mutant			
	HR	Confident Interval low	Confident Interval high	P-value	HR	Confident Interval low	Confident Interval high	P-value	HR	Confident Interval low	Confident Interval high	P-value
	**0.62**	**0.47**	**0.81**	**5.56E-04**	**0.69**	**0.50**		**2.49E-02**	**0.38**	**0.15**	**0.96**	**4.06E-02**
	**2.04**	**1.23**	**3.37**	**5.79E-03**	**2.13**	**1.23**		**7.12E-03**	2.71	0.74	9.92	1.33E-01
**KIAA1429**	**1.71**	**1.15**	**2.53**	**7.64E-03**	1.58	1.00	2.52	5.20E-02	2.24	0.99	5.08	5.36E-02
METTL3	0.73	0.51	1.05	8.91E-02	0.74	0.47	1.16	1.90E-01	1.11	0.42	2.97	8.33E-01
ALKBH5	1.41	0.89	2.26	1.47E-01	1.60	0.95	2.69	7.47E-02	0.78	0.26	2.35	6.64E-01
**RBM15**	1.27	0.91	1.78	1.64E-01	**1.50**	**1.01**	**2.21**	**4.29E-02**	1.16	0.52	2.57	7.16E-01
ZC3H13	1.36	0.86	2.15	1.84E-01	1.13	0.60	2.11	7.06E-01	1.72	0.81	3.63	1.55E-01
YTHDC2	1.35	0.83	2.19	2.29E-01	1.43	0.82	2.49	2.08E-01	1.50	0.51	4.40	4.64E-01
WTAP	1.16	0.90	1.51	2.52E-01	1.03	0.78	1.37	8.12E-01	1.72	0.63	4.70	2.94E-01
HNRNPC	1.25	0.80	1.97	3.32E-01	1.16	0.70	1.90	5.66E-01	2.28	0.79	6.60	1.27E-01
YTHDC1	0.77	0.44	1.35	3.64E-01	1.00	0.49	2.05	9.98E-01	0.82	0.25	2.68	7.46E-01
METTL14	0.85	0.54	1.35	4.94E-01	0.90	0.55	1.46	6.57E-01	0.73	0.18	2.97	6.59E-01
YTHDF1	0.91	0.60	1.39	6.65E-01	0.87	0.56	1.35	5.31E-01	1.14	0.32	4.02	8.41E-01
